# Comparative evaluation of the effect of cold ceramic and MTA-Angelus on cell viability, attachment and differentiation of dental pulp stem cells and periodontal ligament fibroblasts: an in vitro study

**DOI:** 10.1186/s12903-021-01979-1

**Published:** 2021-12-07

**Authors:** Sedigheh Khedmat, Pegah Sarraf, Ehsan Seyedjafari, Parisa Sanaei-rad, Faranak Noori

**Affiliations:** 1 School of Dentistry, Tehran University of Medical Sciences, Tehran, Iran; 2 University of Tehran, Tehran, Iran; 3 School of Dentistry, Arak University of Medical Sciences, Arak, Iran

**Keywords:** Calcium silicate, Cell survival, Cell differentiation, Gene expression, MTA-Angelus, Dental pulp

## Abstract

**Background:**

Biocompatibility and induction of mineralized tissue formation are the properties expected from a material used in vital pulp therapy and repair of perforations. Cold ceramic (SJM, Iran; CC) is a newly introduced calcium silicate-based cement for above mentioned therapeutic applications. This in-vitro study aimed to compare the effect of CC and White MTA-Angelus (MTA) on cell viability, attachment, odontogenic differentiation, and calcification potential of human dental pulp stem cells (DPSCs) and periodontal ligament fibroblasts (PDLFs).

**Methods:**

Cell viability of DPSCs and PDLFs was assessed using MTT on days 1, 3, 7, and 14 (n = 9) in contact with freshly mixed and set states of CC and MTA. Field emission scanning electron micrographs (FESEM) were taken to evaluate cell-bioceramic interaction (n = 6). Gene expression levels of osteo/odontogenic markers (Dentin sialophosphoprotein, Dentin matrix protein 1, Collagen type I alpha 1, and Alkaline phosphatase (DSPP, DMP1, COL 1A1, and ALP, respectively) (n = 8) were assessed using qrt-PCR. ALP enzymatic activity was evaluated to assess the mineralization potential. A two-way ANOVA test was applied, and *p* < 0.05 was considered to be statistically significant.

**Results:**

The effect of freshly mixed and set MTA and CC on the survival of DPSCs and PDLFs in all study groups was statistically similar and comparable to the positive control group (*p* > 0.05); the only exception was for the viability of PDLFs in contact with freshly mixed cements on day 1, showing a more significant cytotoxic effect compared to the control and the set state of materials (*p* < 0.05). PDLFs attached well on CC and MTA. The spread and pseudopodium formation of the cells increased on both samples from day 1 to day 14. Contact of MTA and CC with DPSCs similarly increased expression of all dentinogenesis markers studied on days 7 and 14 compared to the control group (*p* < 0.001), except for DSPP expression on day 7 (*p* = 0.46 and *p* = 0.99 for MTA and CC, respectively).

**Conclusions:**

Within the limitation of this in-vitro study, cold ceramic and MTA-Angelus showed high biocompatibility and induced increased expression of osteo/dentinogenic markers. Therefore, cold ceramic can be a suitable material for vital pulp therapy and the repair of root perforations.

**Supplementary Information:**

The online version contains supplementary material available at 10.1186/s12903-021-01979-1.

## Background

Various materials are used in endodontic treatments to repair root perforations, apexification, and vital pulp therapy. Proper sealability, biocompatibility, and inducing hard tissue regeneration are among the expected properties of a material used in these treatments [1]. Promoting cell attachment and odontogenic differentiation are desirable properties of such materials. These properties not only reflect the biocompatibility of the material but also show that the material may serve as a scaffold for the adjacent cells leading to the construction of mineralized tissues [2]. Calcium silicate-based cements (CSCs) were introduced for the mentioned therapeutic applications. ProRoot MTA (ProRoot MTA, Dentsply, Tulsa, OK) was the earliest member of CSCs [3]. Other commercial bioceramic cements were introduced after introducing this applicable material, such as MTA-Angelus (Angelus, Londrina, Brazil). Despite many advantages, these materials have some drawbacks, such as difficult handling and long setting time [4]. In spite of the manufacturer's claim (15 min), the setting time of MTA Angelus can take approximately 3 h [5]. Moreover, the acidic environment and blood contamination can prolong the setting time even for a week [6]. Cold Ceramic (SJM co, Yazd, Iran; CC) is a newly introduced bioceramic with similar applications of other CSCs, to overcome the shortcomings of previous materials. The radiopacifier of this cement is barium sulfate, which has shown non-toxic properties [7]. According to the manufacturer, the initial and final setting times are 15 min and 24 h, respectively [8]. In a study comparing the effect of blood and saliva contamination on CC and ProRoot MTA, the results showed that the sealing ability of CC is better than MTA in blood-contaminated condition and at least similar to MTA in other conditions [9]. In an animal model, the tissue reaction to CC was comparable to ProRoot MTA, indicating that both materials were biocompatible [10]. Also, an electron scanning microscope (SEM) evaluation revealed that the CC and ProRoot MTA were similar in terms of marginal adaptation [8]. However, the effect of this cement on human cells has not been studied before. The aim of this in-vitro study was to compare the effect of CC and White MTA Angelus (MTA) on cell viability, attachment, odontogenic differentiation, and calcification potential of human dental pulp stem cells (DPSCs) and periodontal ligament fibroblasts (PDLFs).

## Methods

### Preparation of MTA and CC samples

**Fig. 1:**
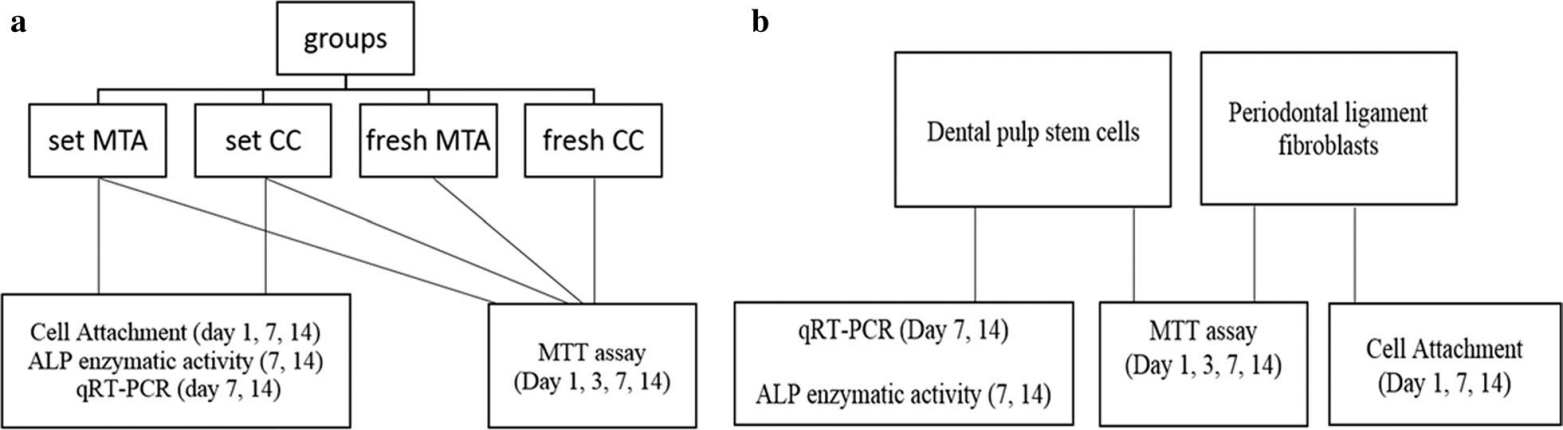
The study design. **a** Experimental groups. **b** Cells used in each assessment

MTA (MTA-Angelus white, Angelus, Londrina, Brazil) and CC (SJM co, Yazd, Iran) were prepared according to the manufacturers' instructions [The components of the cements are provided in Additional file 1]. The samples were cast under the aseptic condition in 10 mm diameter and 1.5 mm height plexiglass mold (Pars, Karaj, Iran). All the samples were exposed to UV light for 20 min on each side. The samples targeted to be evaluated in the set state were incubated for 72 h (to ensure complete setting) at 37 °C and 95% relative humidity. The samples were divided according to the tested materials and target cells. The study design is shown in Fig. 1.

### Cell culture

This study was performed on human dental pulp stem cells (IBRC: C11306) and human periodontal ligament fibroblasts (IBRC: C11326) obtained from the Iranian Biological Resource Center. The cells were cultured in Dulbecco's minimum essential medium (DMEM; Gibco) containing 10% fetal bovine serum (FBS; Sigma) and 100U/ml penicillin, 100 μg/ml streptomycin (Sigma) and incubated at 37 °C in 5% CO_2_.

### Cell viability

The effect of materials on cell viability of DPSCs and PDLFs was assessed using MTT assay (3-(4, 5-dimethylthiazol-2-yl)-2,5-diphenyltetrazolium bromide). The samples were investigated in two states: 1- freshly mixed 2- set. The cytotoxicity assays were conducted in triplicate in order to validate the observed results. In each of these replications, 3 samples of each study group (including MTA-Angelus, Cold Ceramic, and the controls) were prepared and examined. Therefore, a total of 288 samples were examined (n = 9 per group). Both freshly mixed and set groups were transferred to 24-well culture plates containing DMEM for extraction. The area to volume ratio of samples for extraction was according to ISO 10993-5 [11]. Then, the extracts were passed through a 0.22 μm filter. Finally, the extracts were mixed with 10% FBS and 100 IU/ml of penicillin–streptomycin. An equivalent of 100 μl from different extract groups was added to 24-well plates containing DPSCs or PDLFs and culture medium (each plate contained 3.5 × 10^4^ cells/cm^2^). Culture medium without the addition of any extract and distilled water were used as the positive and negative controls, respectively. After 1, 3, 7, and 14 days of incubation, 50 μl of MTT solution was added to the culture medium, and the plates were placed in a wrapped foil in the incubator for 3–4 h. Then the culture medium containing MTT was removed, and 200 μl of Dimethyl sulfoxide (DMSO) was added. Then 100 μl of the above solution was inserted into a 96-well plate. Samples were read at 570 nm wavelength by ELISA reader (Stat Fax 2100, Awareness Technology Inc., Palm City, FL). Cell viability was calculated by dividing the mean absorption of each group by the mean absorption of the positive control group multiplied by 100.

### Cell attachment

This test was performed on PDLFs and the samples in the set state (n = 6). Self-curable glass ionomer (GC Fuji IX, GC Corporation, Tokyo, Japan) was prepared as the control group. 3.5 × 10^4^ cells/cm^2^ were seeded on discs, and DMEM culture medium was added with 10% FBS and 100 IU/ml penicillin–streptomycin. To investigate immediate and long-term cell/surface interactions, field emission scanning electron micrographs (FESEM, Sigma VP, Zeiss, Germany) were taken of the samples on days 1, 7, and 14. The preparation of the samples for FESEM assessment was as follows. The culture mediums of each well were removed, and the samples were washed twice in PBS. Then the cells were fixed with 2.5% glutaraldehyde solution. After 40 min, the samples were rewashed with PBS. The samples were dried in ethanol solution at increasing concentrations (30%, 50%, 70%, 90%, and 100%) for 10 min at each concentration and then air-dried. Conducting process was then performed using the physical vapor deposition (PVD) technique (Coxen, CX-200, South Korea).

### Alkaline phosphatase activity (ALP)

ALP activity of DPSCs was assessed on days 7 and 14. DPSCs (3.5 × 10^4^ cells/cm^2^) were seeded on the set samples of CC and MTA and incubated at 37 °C and 95% relative humidity. After 7 and 14 days of incubation, the surface culture medium was removed, then rinsed with 3 ml PBS. Using 200 μL of RIPA buffer, the enzyme was extracted from stem cells. To eliminate cell debris, the lysate was centrifuged at 15,000 rpm at 4 °C for 20 min. Then, the supernatant was collected, and ALP activity was evaluated with an ALP assay kit (Pars Azmun, Tehran, Iran).

### Osteo/odontogenic differentiation

DPSCs (3.5 × 10^4^ cells/cm^2^) were seeded on the discs of set samples, DMEM was added, and kept in an incubator at 37 °C and 5% CO_2_ for 3 h to establish cell attachment (n = 8). Then the basal medium of each well was replaced with 100 nM dexamethasone, 0.2 mM ascorbic acid 2-phosphate, and 10 mM β-glycerophosphate (Sigma-Aldrich) as differentiation medium. To evaluate the osteo/odontogenic differentiation of DPSCs, the relative expression of specific osteo/dentinogenesis markers, including DMP1, DSPP, COL 1A1, and ALP was measured on days 7 and 14 by the quantitative RT-PCR method. The primer sequences of the examined markers are listed in Table 1. mRNAs were extracted from the cells of study groups using a Hybrid-R™ kit (Gene All, Korea) according to the manufacturer's manual. cDNA synthesis was performed using 1 μg RNA, 0.5 μL dNTPs mix (10 mM), 1 μL of random hexamer primer (10 p.m.), 2 μL reveres transcription buffer, and 0.3 μL RT enzyme (200 U/μL) (Yekta-Tajhis, Iran). Reverse transcription was performed at 25 °C for 10 min and 42 °C for 60 min.

**Table 1:** Primer sequences (Seq. (5–3) of the target differentiation markers

Number	Name	Seq. (5–3)	TM	Per 13–20 µl (total volume) (µl)	Concentration (p/mol)
1	H-beta Actin-F	CTT CCT TCC TGG GCA T	60	1	10
	H-beta Actin-R	GTC TTT GCG GAT GTC CA			
2	DSPP-F	CCA TTC CAG TTC CTC AAA G	60	1	10
	DSPP-R	TGG CAT TTA ACT CAT CCT GTA			
3	ALP-F	GCA CCT GCC TTA CTA ACT	60	1	10
	ALP–R	AGA CAC CCA TCC CAT CT			
4	DMP F	TTCTTTGTGAACTACGGAG	60	1	10
	DMP R	TTGATACCTGGTTACTGGG			
5	H-collagen1α1–F	GCC AAG GGT CTG ACT G	60	1	10
	H-collagen1α1–R	CCC ATC ACA CCA GCC T			

Beta Actin was used as the housekeeping-gene. F, Forward; R, Reverse; concentration used = 10 p/mol, volume = 1 µl, melting temperature (Temperature melting (TM) = 60 °C

Quantitative real-time PCR reactions were prepared in a final volume of 20 μL containing 1 μL of each primer (forward and reverse, 10 p.m.), 1 μL cDNA, and 10 μL SYBR® Premix Ex Taq ™ II (Perfect Real Time; Takara, Dalian, China). StepOne™ Software v2.2.2 analyzed threshold cycles (Cq). The relative expression of target genes was determined using the ΔΔCT method and REST® 2009.

### Statistical analysis

Statistical analyses were performed using GraphPad Prism software, Version 6 (GraphPad Software, San Diego CA, USA). Mean ± standard deviation (SD) was calculated [Additional file 2], and a two-way ANOVA test was applied for analyzing the differences. P-values less than 0.05 were considered to be statistically significant.

## Results

### Cell viability

**Fig. 2:**
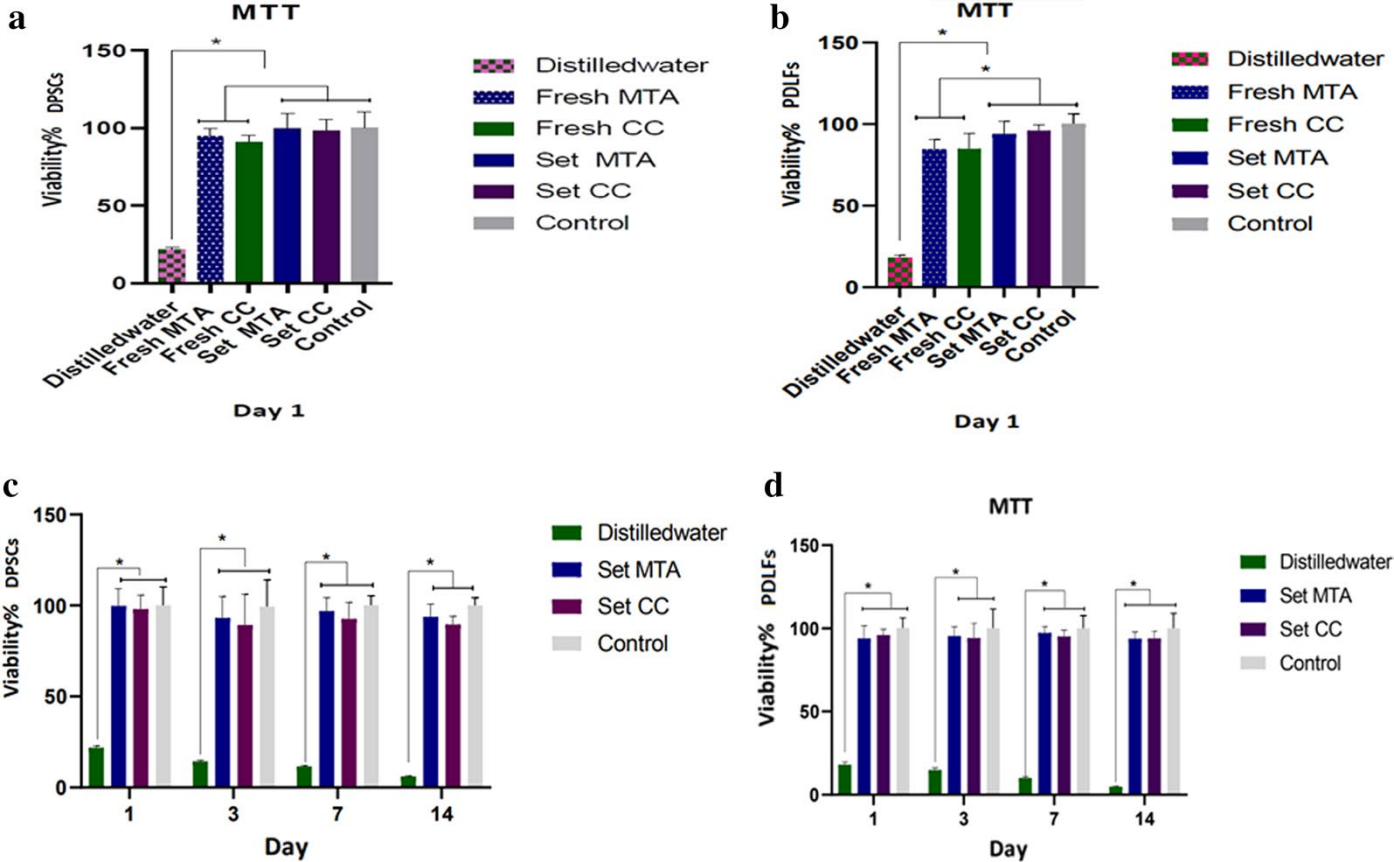
The results of the MTT assays. **a** comparison of DPSCs viability after exposure to freshly mixed and set materials on day 1. **b** comparison of PDLFs viability after exposure to freshly mixed and set materials on day 1, indicating a more significant cytotoxic effect of freshly mixed of both materials compared to the control group and the set state of substances (*p* < 0.05). **c** and **d** DPSCs and PDLFs viability after exposure to set state of materials for 1, 3, 7, and 14 days showed no significant difference among the study groups and the positive control (*p* > 0.05). * Indicates statistically significant difference

The results of the MTT assays are shown in Fig. 2. The effects of freshly mixed and set MTA and CC on the viability of DPSCs and PDLFs in all time points were statistically similar and comparable to the positive control group (*p* > 0.05). The only exception was the effect of freshly mixed CC and MTA on PDLFs on day 1, which showed a significant cytotoxic effect compared to the positive control group (*p* < 0.01) and the set state of the samples (*p* = 0.0198 and *p* = 0.0046 for MTA and CC, respectively). Distilled water (negative control) showed a more cytotoxic effect in comparison to the other groups in all of the studied time points (*p* < 0.001).

### Cell attachment

**Fig. 3:**
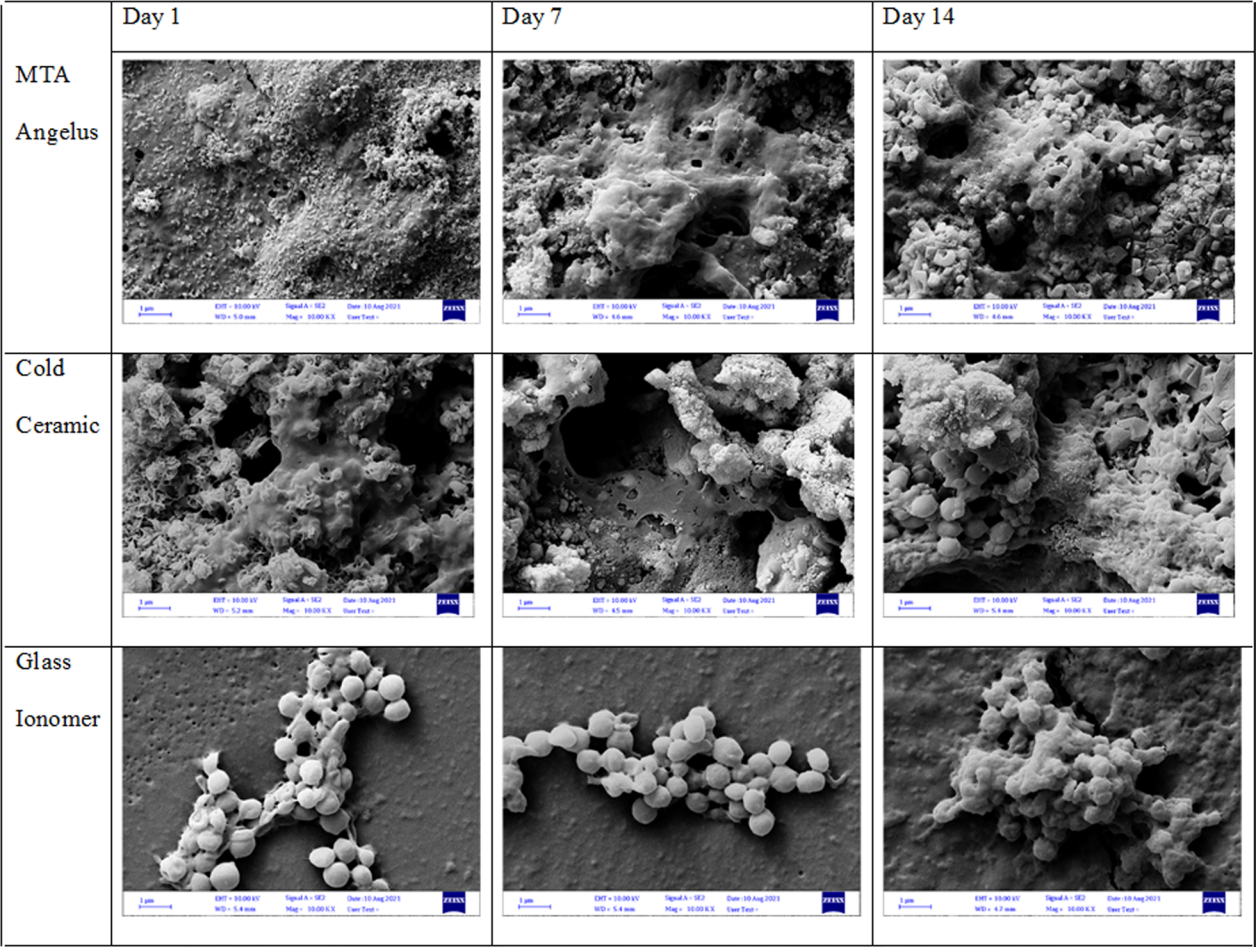
A field emission scanning electron micrograph (FESEM) of PDLFs seeded on MTA, CC, and GI (magnification 10,000 ×). Cells attached well and similarly on MTA and CC; however, cell attachment on GI samples was poor

As shown in Fig. 3, PDLFs attached favorably on both CC and MTA samples. The spread and pseudopodium formation of the cells increased on both samples over time from day 1 to day 14. Cell attachment was observed on GI samples. However, demonstration of round-shaped cells and poorly branched processes indicated poor cell attachment.

### ALP activity

**Fig. 4:**
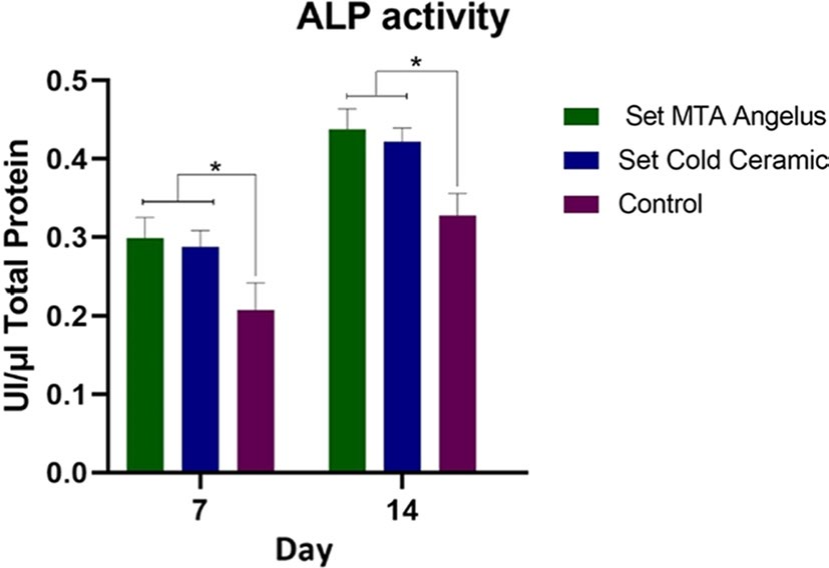
ALP activity of DPSCs in direct contact with MTA and CC. There were no significant differences between CC and MTA groups on days 7 and 14. Both of the materials significantly increased ALP activity of DPSCs compared to the control (*p* < 0.001). Values are expressed as the total number of ALP enzymes per μl. * Indicates statistically significant difference

The results of the ALP activity are shown in Fig. 4. The enzymatic activity of DPSCs was significantly higher when they were in contact with CC and MTA compared to the control in all time intervals (*p* < 0.001). Cells in contact with MTA groups showed higher ALP activity in all time points compared to CC, but this difference was not statistically significant (*p* = 0.26).

### q RT-PCR

**Fig. 5:**
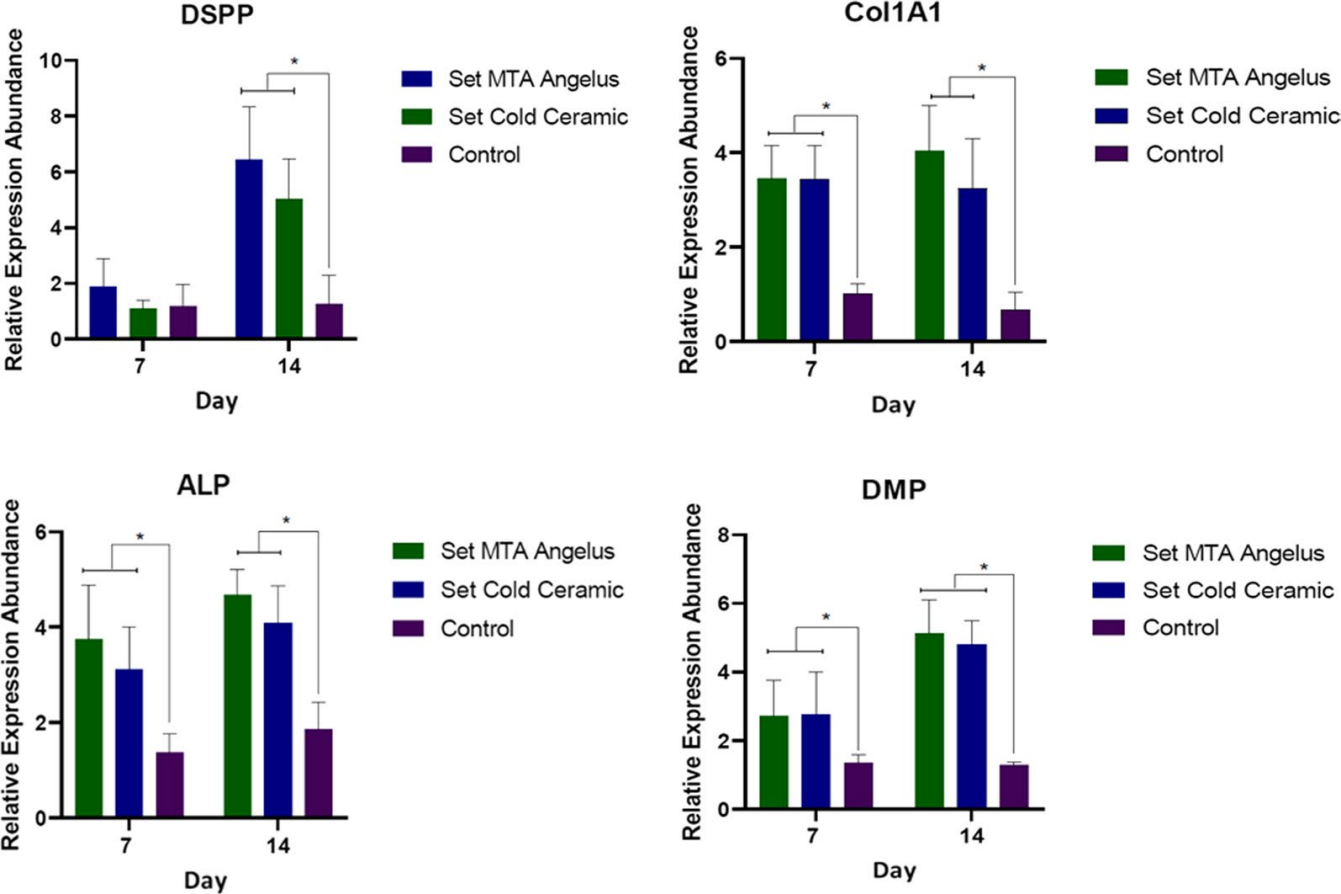
Relative expression of target odontogenic markers. MTA and CC increased the expression of odontogenic markers by DPSCs, except in DSPP expression on the 7-day time interval. * Indicates statistically significant difference

The results of the relative expression of target odontogenic markers are shown in Fig. 5. MTA and CC contact with DPSCs resulted in an increased expression of dentinogenesis markers including DSPP, DMP1, COL 1A1, and ALP compared to the control group on days 7 and 14 (*p* < 0.001). The only exception was DSPP gene expression on day 7 of contact with MTA and CC samples, which was similar to the control (*p* = 0.46 and *p* = 0.99, respectively). However, these values were significantly higher than the control on day 14 (*p* < 0.001). There was no significant difference between CC and MTA samples regarding time periods and markers expression (*p* > 0.05).

## Discussion

This in-vitro study aimed to evaluate and compare certain biological effects of CC and MTA on DPSCs and PDLFs. Overall, both cements showed high biocompatibility and favorable cell attachment, and also induced increased expression of osteo/odontogenic differentiation markers.

To evaluate the biocompatibility of a material in direct contact with vital tissues, various tests such as the assessment of cytotoxicity are compulsory [12]. Various methods and cell lines were used in previous studies to examine the biocompatibility of CSCs [13–15]. Also, some studies have been examined different concentrations of the studied materials (i.e., serial dilution method) [2, 16]. Despite providing comprehensive information about the studied subjects and compliance with laboratory standards, these concentrations may differ from those used in biological conditions and clinical applications. Therefore, it is necessary to interpret the results of these studies with caution. Also, due to these variations in methods, target cells, and concentrations of tested materials, it is impossible to compare the results of different studies reliably. In the present study, the ISO-10993-5 standard was used to evaluate the cytotoxic effect of the studied materials. This standard describes the necessary tests, protocols, and standard concentrations to evaluate the biological effects of medical substances in contact with body tissues in a laboratory setting [11]. Due to the standardization, it is easier to compare the results of this study with similar studies accurately and interpret the result for clinical situations.

DPSCs and PDLFs are in direct contact with the applied sealing material in vital pulp therapy and repair of perforations. Therefore, to simulate clinical conditions, these cells were selected for the present study.

MTT has been shown as a reliable method for in-vitro cytotoxicity evaluation of materials used in endodontics [17]. Previous studies indicated that MTA Angelus has high biocompatibility [16, 18, 19]. While the results of one study showed that cell proliferation increases in the presence of MTA Angelus [16], another study have indicated that its effect is similar to that of the control group (plain culture medium) [20]. The difference in the results of these studies could be due to the use of different cell lines (DPSCs or fibroblasts), the extraction protocol, and the concentrations of the extract. As mentioned earlier, the use of ISO-10993 makes it possible to interpret and compare the results of in-vitro studies more accurately.

In the clinical application, CSCs are placed on living tissue immediately after mixing. Therefore, in-vitro assessment of the cements in the freshly mixed state seems necessary to predict the initial response of the tissues. Furthermore, evaluation of the set state of the cements may indicate the long-term response of cells and tissues to the materials [21].Therefore, in this study, the possible cytotoxicity of the eluates from both freshly mixed and set states of the cements were evaluated..

The results of the present study showed that the biocompatibility of CC in freshly mixed and set states at all time points studied was statistically similar to MTA Angelus. The cell viability in all groups was similar to the positive control group. The only exception was the effect of freshly mixed materials on PDL fibroblasts, which was lower than the control, which could be attributed to the alkaline pH of the freshly mixed cements [22]. The difference in cell reaction may be related to the growth rate of the cells [23, 24]. The high growth rate of DPSCs may have compensated for the toxicity of the samples, but this compensation did not occur in fibroblasts due to the slower growth rate compared to DPSCs.

According to ISO-10993-5, substances that reduce cell viability by more than 30% are considered cytotoxic [25]. None of the study groups showed a mean survival rate of less than 89% in the present study. Therefore, it can be concluded that CC and MTA are biocompatible in both fresh and set states.

Another method to assess the biocompatibility of a material is to evaluate the ability of a selected cell line to attach to the material's surface (16). Various factors affect cell-substance interaction, including the surface porosity and roughness of the material [26]. The surface roughness promotes cell attachment and proliferation [27]. The results of the present study showed that PDLFs could properly attach on the surfaces of both CC and MTA cements. Cell spreading, intercellular communication, and pseudopodia formation were observed on both studied cements over time. This finding was similar to the previous studies on different cell-bioceramic interactions [28, 29].

Alkaline phosphatase (ALP) plays an essential role in the initial formation of mineralized tissues and induction of hydroxyapatite deposition in collagenous matrices. This enzyme stimulates dentin matrix formation by pulp tissue; hence, it is essential for healing and repairing after pulpal injury [30]. The results of the present study showed that CC and MTA both could increase ALP enzymatic activity. This finding was in accordance with previous studies [28, 31]. Therefore, it may be assumed that the contact of CC and MTA with DPSCs leads to increased mineralization activity of these cells. This finding is important because it demonstrates the potential of these materials to induce dentin bridge formation in vital pulp therapy.

CSCs induce mesenchymal stem cells differentiation by creating an osteo/odontogenic inductive medium [32, 33]. The hydrolysis of the cements and production of calcium hydroxide might be the reason for this bioactive property [22]. The result of this cell differentiation is an increase in the expression of specific mineralization genes. DSPP, DMP1, COL 1A1, and ALP are among the most significant dentinogenesis markers [28]. In this study, the mentioned genetic markers were used to evaluate the odontogenic differentiation of DPSCs. In 2015 a review study by Rathinam et al. [34] found that various bioceramics including Biodentine, MTA Angelus, OrthoMTA, MicroMega MTA, Portland cement, Endocem, and ProRoot MTA increased osteo/odontogenic markers expression similarly. On the other hand, other cements such as iRoot BP Plus, Bioaggregate, and CEM increased the expression of those markers more than ProRoot MTA. The reasons for the difference between different tricalcium silicate-based cements have not been identified so far [34]. However, differences in setting time, initial and final pH of the cements, the rate of calcium ion release [21, 35], as well as the type of culture medium used in the control group (conventional nutrient culture medium versus differentiation medium) may be the justification for the differences in relative expression. The results of the present study showed that both MTA and CC increased the expression of DSPP, DMP1, COL 1A1, and ALP markers similarly, which was higher compared to the control group. However, this increase was not observed for DSPP expression on day 7. Though, with the increase of exposure time to 14 days, the expression of this marker increased in both CC and MTA and was higher than the control group. These results indicate that the application of MTA and CC in vital pulp therapy not only provides a proper seal for the exposed pulp tissues [9, 36] but also stimulates odontogenic differentiation of the DPSCs resulting in dentinal bridge formation.

The present study investigated certain biological effects of two types of CSCs on the target cells in vital pulp therapy and root perforation repair, in a laboratory setting. However, in-vivo studies are recommended to better investigate and generalize the effects of these biomaterials in the clinical practice.

## Conclusions

Within the limitation of this in-vitro study, cold ceramic and MTA-Angelus have high biocompatibility. These materials increase the expression of osteo/odontogenic differentiation markers. Also, Cell attachment occurs favorably on both of these materials. Therefore, cold ceramic can be a suitable material for vital pulp therapy and repair of root perforations.

## Supplementary Information

**Additional file 1**. The chemical components of MTA Angelus and Cold Ceramic cements.

**Additional file 2**. The datasets generated and analyzed during the current study.

## Data Availability

All data generated or analyzed during this study are included in this article as "Additional file 2" in the Statistical analysis part of the method section.

## Abbreviations

DPSCs: Dental pulp stem cells
PDLFs: Periodontal ligament fibroblasts
MTA: MTA-Angelus
CC: Cold ceramic
DMP1: Dentin matrix protein 1
DSPP: Dentin sialophosphoprotein
COL 1A1: Collagen, type I, alpha 1
ALP: Alkaline phosphatase
