# Supplementary material for: Comparative evaluation of the effect of cold ceramic and MTA-Angelus on cell viability, attachment and differentiation of dental pulp stem cells and periodontal ligament fibroblasts: an in vitro study
Source: BMC Oral Health. 2021 Dec 7;21:628. doi: 10.1186/s12903-021-01979-1 (PMC8650362; doi:10.1186/s12903-021-01979-1)
Supplement: Supplementary file 1 — Additional file 1. The chemical components of MTA Angelus and Cold Ceramic cements. [file 12903_2021_1979_MOESM1_ESM.docx]

Additional file 1. The chemical components of MTA Angelus and Cold Ceramic cements.

Table 1: Chemical components of the studied materials. + Not detected

| Cold Ceramic | MTA Angelus | Materials  Component |
| --- | --- | --- |
| 48.12 | 49.20 | CaO |
| 16.19 | 18.58 | SiO_2_ |
| ND^+^ | 8.26 | Bi_2_O_3_ |
| 18.61 | ND^+^ | BaO |
| ND^+^ | 4.48 | Al_2_O_3_ |
| 0.39 | 0.64 | MgO |
| 0.36 | ND^+^ | FeO |
| 10.15 | 0.19 | SO_3_ |
| ND^+^ | ND^+^ | P_2_O_5_ |
| 0.10 | ND^+^ | TiO_2_ |
| 0.08 | 1.32 | Na_2_O |
| ND^+^ | 0.51 | Cl |
