# Supplementary material for: Comparative evaluation of the effect of cold ceramic and MTA-Angelus on cell viability, attachment and differentiation of dental pulp stem cells and periodontal ligament fibroblasts: an in vitro study
Source: BMC Oral Health. 2021 Dec 7;21:628. doi: 10.1186/s12903-021-01979-1 (PMC8650362; doi:10.1186/s12903-021-01979-1)
Supplement: Supplementary file 2 — Additional file 2. The datasets generated and analyzed during the current study. [file 12903_2021_1979_MOESM2_ESM.docx]

Additional file 2. The datasets generated and analyzed during the current study.

Table 1- Mean and standard deviation (SD) values of cell viability in MTT assay. Positive control: DMEM. Negative control: Distilled water. f: Fresh. s: Set. MTA: MTA-Angelus. CC: Cold Ceramic. hDPSC: human dental pulp stem cell. hPDLF: human periodontal ligament fibroblast.

| Time | material | Cell line | Mean | SD |
| --- | --- | --- | --- | --- |
|  |  |  |  |  |
| 1 day | Positive Control | hDPSC | 100.0000 | 10.15092 |
|  | Negative control | hDPSC | 21.8536 | 0.96094 |
|  | f.MTA | hDPSC | 94.8920 | 4.63013 |
|  | f.CC | hDPSC | 90.8373 | 4.52104 |
|  | s.MTA | hDPSC | 99.7894 | 9.38374 |
|  | s.CC | hDPSC | 98.0516 | 7.68754 |
| 3 days | Positive Control | hDPSC | 99.4872 | 14.55209 |
|  | s.MTA | hDPSC | 93.1966 | 11.73317 |
|  | s.CC | hDPSC | 89.2650 | 16.95413 |
| 7 days | Positive Control | hDPSC | 100.1944 | 5.16868 |
|  | s.MTA | hDPSC | 96.9722 | 7.32552 |
|  | s.CC | hDPSC | 92.6389 | 9.06927 |
| 14 days | Positive Control | hDPSC | 100.0726 | 4.26498 |
|  | s.MTA | hDPSC | 93.8707 | 6.94087 |
|  | s.CC | hDPSC | 89.5134 | 3.22042 |
| 1 day | Positive Control | hPDLF | 100.3419 | 6.02155 |
|  | Negative control | hPDLF | 18.2051 | 1.46457 |
|  | f.MTA | hPDLF | 84.6154 | 5.96154 |
|  | f.CC | hPDLF | 85.0000 | 9.35612 |
|  | s.MTA | hPDLF | 94.1026 | 7.55405 |
|  | s.CC | hPDLF | 96.0684 | 3.44428 |
| 3 days | Positive Control | hPDLF | 100.2110 | 11.53654 |
|  | s.MTA | hPDLF | 95.3938 | 5.51331 |
|  | s.CC | hPDLF | 94.1632 | 8.80565 |
| 7 days | Positive Control | hPDLF | 100.0428 | 7.60394 |
|  | s.MTA | hPDLF | 97.3239 | 3.80929 |
|  | s.CC | hPDLF | 95.2259 | 3.68209 |
| 14 days | Positive Control | hPDLF | 100.0890 | 8.93803 |
|  | s.MTA | hPDLF | 93.8049 | 4.09201 |
|  | s.CC | hPDLF | 94.0496 | 4.11085 |

Table 2- Mean and standard deviation (SD) values of ALP activity assessment. MTA: MTA-Angelus. CC: Cold Ceramic.

| Time | Material | Mean | SD |
| --- | --- | --- | --- |
|  |  |  |  |
| 7 days | Control | 0.2075 | 0.03433 |
|  | MTA | 0.2989 | 0.02643 |
|  | CC | 0.2875 | 0.02087 |
| 14 days | Control | 0.3278 | 0.02784 |
|  | MTA | 0.4378 | 0.02631 |
|  | CC | 0.4219 | 0.01736 |

Table 3- Mean and standard deviation (SD) values of relative gene expression in qrt-PCR assessment. MTA: MTA-Angelus. CC: Cold Ceramic.

| Targeted gene | Time | Material | Mean | SD  (Standard Deviation) |
| --- | --- | --- | --- | --- |
| COL 1A1 | 7 days | Control | 1.019249 | 0.197084 |
|  |  | MTA | 3.448665 | 0.705332 |
|  |  | CC | 3.448665 | 0.705332 |
|  | 14 days | Control | 0.683735 | 0.365448 |
|  |  | MTA | 4.04642 | 0.960464 |
|  |  | CC | 3.245441 | 1.0516 |
| DSPP | 7 days | Control | 1.030628 | 0.285519 |
|  |  | MTA | 1.871818 | 1.018957 |
|  |  | CC | 1.102988 | 0.289304 |
|  | 14 days | Control | 1.249029 | 1.039245 |
|  |  | MTA | 6.44583 | 1.884957 |
|  |  | CC | 5.041591 | 1.428964 |
| DMP1 | 7 days | Control | 1.358304 | 0.22697 |
|  |  | MTA | 2.725537 | 1.039556 |
|  |  | CC | 2.760885 | 1.231813 |
|  | 14 days | Control | 1.570087 | 0.409738 |
|  |  | MTA | 5.117907 | 0.988722 |
|  |  | CC | 4.795675 | 0.709349 |
| ALP | 7 days | Control | 1.36763 | 0.393595321 |
|  |  | MTA | 3.754343 | 1.134136 |
|  |  | CC | 3.111612 | 0.899709 |
|  | 14 days | Control | 1.866947 | 0.566366 |
|  |  | MTA | 4.681449 | 0.528205 |
|  |  | CC | 4.094461 | 0.776857 |
